# A rare case of synovial sarcoma of diaphragm

**DOI:** 10.1002/cnr2.1622

**Published:** 2022-04-29

**Authors:** Anand Kumar Mishra, Javid Raja, Apeksha Mittal, Vidur Bansal

**Affiliations:** ^1^ Department of Cardiothoracic and Vascular Surgery Post Graduate Institute of Medical Education and Research Chandigarh India

**Keywords:** diaphragm, mesh reconstruction, synovial sarcoma

## Abstract

**Background:**

Primary diaphragmatic synovial sarcoma is a rare clinical entity with only few cases reported in the literature. It is found mainly in young adults, in the limbs. However, the name is a misnomer as it probably arises from primitive mesenchyme rather than articular surfaces of the joints.

**Case:**

We report a case of 21‐year‐old patient with synovial sarcoma of the diaphragm, treated by complete surgical excision of the tumor with diaphragmatic reconstruction and confirmed on immunohistopathology. The peculiarity of this case stems from the atypical location of the tumor with complete surgical resection and thereby providing a better quality of life for the patient.

**Conclusion:**

Synovial sarcoma of the diaphragm is a rare malignancy and more data and research is needed for defining the best management for this tumor.

## 1 INTRODUCTION

Synovial sarcoma is a rare but highly malignant mesenchymal tumor accounting for approximately 5%–10% of all soft tissue sarcomas, mainly found inadolescents and young adults.^1^ Majority of synovial sarcomas arise near articular surfaces especially of lower limbs. However, the name is a misnomer, as the histogenesis of these lesions is still debated with some evidence of origin from primitive mesenchyme.^2^ Complete surgical excision of the tumor remains the first line of therapy.^3^ Here, we report a synovial sarcoma of the diaphragm with radical surgical excision and reconstruction of the diaphragm.

## 2 CASE REPORT

**FIGURE 1:**
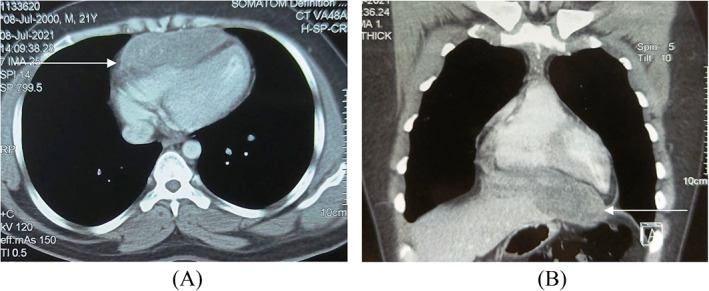
Contrast enhanced CT of chest (A) axial and (B) coronal images showing a heterogeneously hyperdense mass in relation to inferior surface of heart and diaphragm (arrow indicates the mass).

A 21‐year‐old male with no significant medical history, presented with complaints of chest pain and dyspnoea on exertion for 6 months. The patient had no constitutional symptoms or family history of malignancy. Electrocardiogram was suggestive of sinus tachycardia and global T wave inversion. Chest x‐ray was grossly normal. Echocardiography revealed large hypoechoic pericardial mass measuring 6.5 x 3 cm abutting the right ventricular free wall causing compression of the right ventricle. Contrast Enhanced computed tomography of the chest showed a well‐defined heterogenously hyperdense non‐enhancing mass in relation to pericardium, along the inferior aspect of heart, causing indentation of the right ventricle with preservation of pericardial fat plane (Figure 1).

**FIGURE 2:**
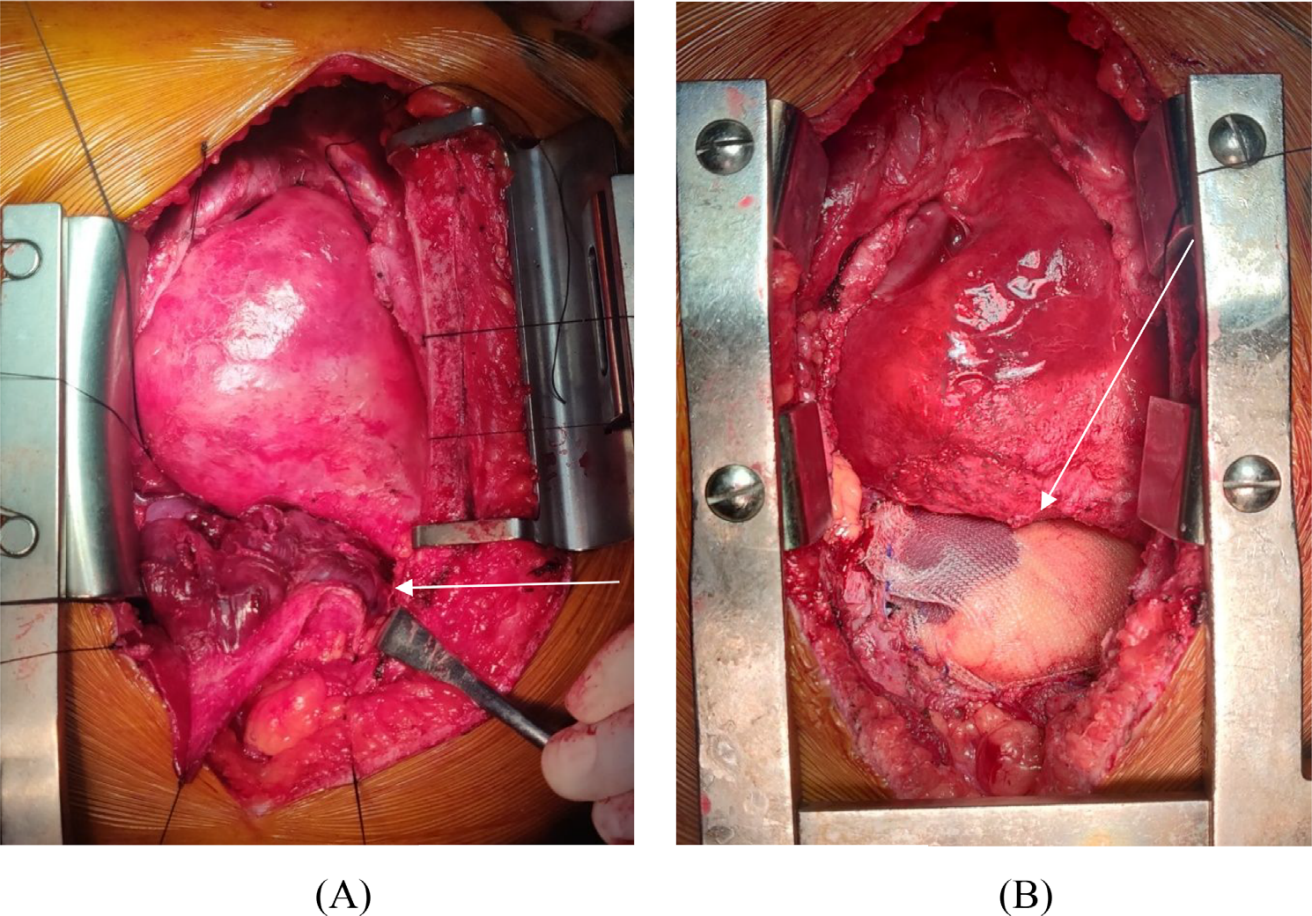
Intraoperative images showing (A) tumor in situ (arrow), in relation with right ventricle and diaphragm; (B) mesh reconstruction of diaphragmatic defect (arrow poiting to the mesh).

Complete surgical excision of the lesion was planned for both diagnostic and therapeutic purposes. The approach was through median sternotomy. The pump was prepared to go on cardiopulmonary bypass whenever needed. Intra‐operatively tumor was found arising from superior surface of diaphragm, abutting the anterior surface of right ventricle and left ventricle apex, and adjacent pericardium. Frozen section biopsy was done and was suggestive of sarcoma. So, radical excision of the tumor was undertaken and the tumor was excised along with the involved pericardium starting from the right ventricle and then the diaphragm with a clear macroscopic margin and the defect in diaphragm was repaired with prolene mesh (Figure 2). The postoperative period went uneventful. Histopathological examination revealed spindle shaped tumor cells arranged in sheets and long fascicles with areas of hemorrhage and necrosis with negative margins. Immunohistochemistry showed patch positivity for CD99 and diffuse nuclear positivity for TLE‐1 suggestive of monophasic spindle cell synovial sarcoma, FNCLCC grade 3. It was staged as AJCC stage III‐ B and currently the patient is undergoing adjuvant chemo‐radiotherapy.

## 3 DISCUSSION

Synovial sarcoma, represents a rare type of soft tissue sarcoma (STS) of uncertain differentiation, accounting for 5%–10% of all STS. Compared to other STS, it presents at a younger age and has equal predilection for both sexes.^1^ Most commonly affected sites include extremities, especially lower extremities, but have also been reported from head and neck, lung, pleura, mediastinum, abdominal wall, mesentery and esophagus.^4^ Synovial sarcoma of thorax can present with chest pain, dyspnoea, cough, hemoptysis, pleural or pericardial effusions.^5^

Diagnosis is usually hinted by trans‐thoracic echocardiography and it can also determine the location, size and functional impact of the tumor to a limited extent.^6^ Computed tomography and magnetic resonance imaging helps identify the location of the tumor, it's extent and invasion of surrounding organs. These are essential for surgical planning. MRI helps better delineate the soft tissue planes and is a better modality over CT for surgical planning.^7^, ^8^ In the index case, initial diagnosis was suspected on echocardiography with computed tomography helped us in planning the management.

Primary synovial sarcoma can be monophasic or biphasic or poorly differentiated depending on relative abundance of spindle and epithelial cells, nuclear atypia and mitotic figures. On immunohistochemistry, 60% of these tumors stain positive for CD99 and diffuse expression of bcl‐2 can be seen. Immunohistochemistry also demonstrates strong and diffuse nuclear staining for transcriptional co‐repressor TLE1.^9^ In the present case, the synovial sarcoma was monophasic spindle cell type with CD99, bcl‐2 and TLE‐1 positivity.

Radical surgical excision remains the first line treatment for these patients.^10^, ^11^ In cases of extremity synovial sarcoma neoadjuvant or adjuvant radiation therapy is recommended for larger tumors (>5 cm), or in cases where a close margin may be required to preserve a major neurovascular structure or bone. Chemotherapy is reserved for patients with high‐risk tumors or advanced disease.^8^ However due to paucity of cases, optimal treatment strategies for intra‐thoracic synovial sarcoma like in this reported case are yet to be defined. The indexed case is undergoing adjuvant chemo‐radiotherapy as decided by a multi‐disciplinary team to reduce chances of recurrence. High grade tumors like these have a high risk of recurrence, however an R0 resection coupled with adjuvant therapy in this patient may lead to improved outcomes. As there is a very limited literature regarding the case discussed here it becomes difficult to determine the exact prognosis and surveillance plan for this patient. As there are no tumor markers, we plan on doing 3 monthly clinical evaluation, echocardiogram and chest x‐ray and a CECT chest after 6 months.

## 4 CONCLUSION

Synovial sarcoma of diaphragm is a rare malignancy and multidisciplinary approach is needed for its management. However, due to paucity of data, optimal treatment strategies are yet to be defined and more research is needed in this regard.

## CONFLICT OF INTEREST

The authors declare no conflict of interest.

## AUTHOR CONTRIBUTIONS

All authors had full access to the data in the study and take responsibility for the integrity of the data. *Conceptualization*, A.K.M.; *Methodology*, J.R., V.B.; *Writing—Original Draft*, A.M.; *Supervision*, A.K.M.; *Project Administration*, A.M., J.R.

## ETHICAL STATEMENT

This manuscript was approved by the institutional ethics committee. Informed consent was taken from the patient.

## Data Availability

The data regarding this study can be availed from the corresponding author upon request.
